# An eDNA Survey of Plant Biodiversity in a Local Dam Within South Africa's Largest City

**DOI:** 10.1002/ece3.72196

**Published:** 2025-09-28

**Authors:** N. Nhlengethwa, R. D. Stewart, A. Emami‐Khoyi, P. R. Teske, S. Csányi, M. Heltai, M. van der Bank

**Affiliations:** ^1^ African Centre for DNA Barcoding, Department of Botany and Plant Biotechnology University of Johannesburg Auckland Park South Africa; ^2^ Department of Biological & Agricultural Sciences Sol Plaatje University Kimberley South Africa; ^3^ Institute for Wildlife Management and Nature Conservation, Department of Wildlife Biology and Management Hungarian University of Agriculture and Life Sciences Gödöllő Hungary; ^4^ Centre for Ecological Genomic and Wildlife Conservation University of Johannesburg Auckland Park South Africa

**Keywords:** biodiversity, DNA barcoding, environmental DNA (eDNA), freshwater, invasive species, metabarcoding, South Africa, urban ecosystem

## Abstract

Ecosystems within cities can play a crucial role in conserving local biodiversity amid rapidly expanding urban sprawl, but they face significant threats from anthropogenic activities and the introduction of alien invasive species (AIS). A comprehensive management plan is required to effectively preserve the biodiversity supported by urban ecosystems. However, the ecological information needed to establish, implement and monitor such plans is often incomplete. In this study, we assessed the application of eDNA metabarcoding in surveying plant biodiversity in an aquatic habitat by collecting water samples at five sites in an urban dam in the City of Johannesburg. Out of 1001 reconstructed Amplicon Sample Variants (ASVs), plant taxa were assigned to 47 unique taxonomic ranks at the family level, 42 unique ranks at the generic level and only 13 unique ranks at the species level (including three AIS). The remaining ASVs could only be identified at higher taxonomic ranks, indicating that no DNA barcodes have yet been generated for the putative species in question. Although this study provides a good overview of plant community structure, it also highlights a gap in the taxonomic coverage of South African plants on public DNA databases. To address this shortcoming, increased national DNA barcoding efforts are needed to expand current reference databases. This will be indispensable for the effective application of eDNA metabarcoding in studying South Africa's unique biodiversity.

## Graphical abstract

Ecological information is needed to establish, implement and monitor such biodiversity management plans. This study employs environmental DNA to provide a good overview of plant community structure in an urban dam in Johannesburg, South Africa. It further reveals a gap in the taxonomic coverage of South African plants on public DNA databases.

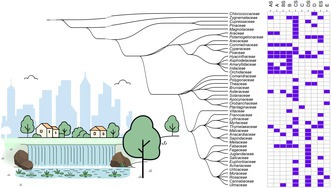

## 1 Introduction

South Africa is one of the most biologically diverse countries on Earth. Its unique bioclimatic, oceanographic, geological and topographical features make it one of the top 10 megadiverse countries in terms of plant biodiversity (Klopper 2010; Tolley et al. 2019; Mamathaba et al. 2022). South Africa is home to 3 of the 36 world's biodiversity hotspots (Klopper 2010; Mamathaba et al. 2022), and its rich biodiversity is characterised by a high level of species richness and endemism in various ecosystems. The National Biodiversity Assessment (NBA) of 2019 revealed that the country has over 20,401 described plant species, 66% of which are endemic (Tolley et al. 2019). This unique biodiversity, however, has been negatively impacted by urban expansion, conversion of natural ecosystems to agricultural lands, plantation forestry and industrial mining. Skowno et al. (2021) suggest that the country has lost approximately 22% of its natural habitat over the past century.

Rapid and unplanned urban expansion has historically been associated with the loss of wild habitat and is considered one of the primary causes of species extinction (WWF 2016; Abell et al. 2019; Petersen et al. 2023). Nevertheless, effectively managed urban ecosystems can provide niches for the persistence of local species (Adams 2016) when urban sprawl encroaches on formerly pristine habitats and may preserve a significant amount of biodiversity (Abell et al. 2019; Petersen et al. 2023; Oladimeji et al. 2024).

The present study surveyed plant biodiversity in a dam in the City of Johannesburg, South Africa's largest metropolitan area. The city's population is predicted to increase significantly over the next few decades as people from rural areas migrate to urban spaces (WWF 2016; Abell et al. 2019; Petersen et al. 2023). The resulting encroachment of urban development into the natural habitats surrounding the city poses a significant threat to local biodiversity (Gauteng Department of Agriculture, Rural Development, and Environment 2022).

To mitigate these adverse impacts, a comprehensive urban ecosystem management plan is required. However, insufficient ecological data, exacerbated by poor knowledge of aquatic and terrestrial ecosystem types and their current levels of biodiversity, present major challenges to the establishment and implementation of policies that regulate the sustainable management of biodiversity in urban areas (Department of Water and Sanitation (DWS) 2016; Skowno et al. 2019).

Aquatic environments are crucial in cities. In addition to serving as aggregation points for species living in adjacent habitats, they provide migration corridors and stepping stones for species dispersal, and they play an essential role in the health of the ecosystem as a whole (Calapez et al. 2023). Yet, they face a significant threat from water abstraction, pollution and the introduction of alien invasive species (AIS). Some AIS may cover entire ecosystems, forming dense mats of vegetation that block out the sunlight and prevent indigenous plants from growing, thus reducing the proper ecological functioning of these habitats. This, in turn, results in an increase in flood risks and reduces aquatic biodiversity (Hill et al. 2020; Chamier et al. 2012). The resulting ecologically impoverished ecosystems may then serve as reservoirs for the proliferation of various pathogens and pose a health risk to the people living in their vicinity (Havel et al. 2015; Ngobeni 2020).

The presence or absence of specific plant assemblages in an ecosystem can be used to diagnose its current ecological state (Delmail 2014). Various methods have been used to monitor the health of ecosystems, including assessments of bioaccumulation, biochemical alterations, behavioural and morphological observations, as well as ecological surveys and modelling (Michalak and Chojnacka 2014). These methods are labour‐intensive and depend on the morphological identification of indicator species, requiring trained taxonomic experts for specimen collection, preparation and identification (Rouhan and Gaudeul 2021).

Recent advances in molecular methods and the application of environmental DNA (eDNA) in ecological studies provide an efficient method of monitoring biodiversity at the community level, without the need to visually identify species (Ruppert et al. 2019; Beng and Corlett 2020; Nagarajan et al. 2022; Yao et al. 2022; Rishan et al. 2023). Trace amounts of DNA shed by organisms into the environment have been successfully retrieved and sequenced to monitor various aquatic and terrestrial ecosystems (Thomsen and Willerslev 2015; Webster et al. 2020; Yao et al. 2022; Emami‐Khoyi et al. 2025). By identifying matches with DNA sequences lodged in the reference sequence databases, the amplification of the eDNA originating from entire biological communities can be used to identify the species living in a particular ecosystem (Coghlan et al. 2021).

Freshwater dams, ponds and pools play an essential role as accumulation media for the successful retrieval of both autochthonous and allochthonous eDNA molecules (Harper et al. 2019). They act as natural reservoirs for eDNA originating from adjacent habitats that are transported by wind, stormwater drainage and precipitation. In addition, aquatic environments protect the DNA molecules from UV light and extreme temperature fluctuations through their higher turbidity and dense vegetation cover, facilitating the accumulation of eDNA molecules over time (Harper et al. 2019; Bozdogan et al. 2025).

Here, we present the first metabarcoding survey of plant biodiversity in an urban freshwater habitat in South Africa. It serves as an initial step toward better understanding the unexplored biodiversity in urban ecosystems and represents a proof‐of‐concept that demonstrates the strengths of using molecular methods in documenting biodiversity, while also highlighting challenges that are unique to conducting such studies in developing nations.

## 2 Materials and Methods

### 2.1 Study Area

**FIGURE 1:**
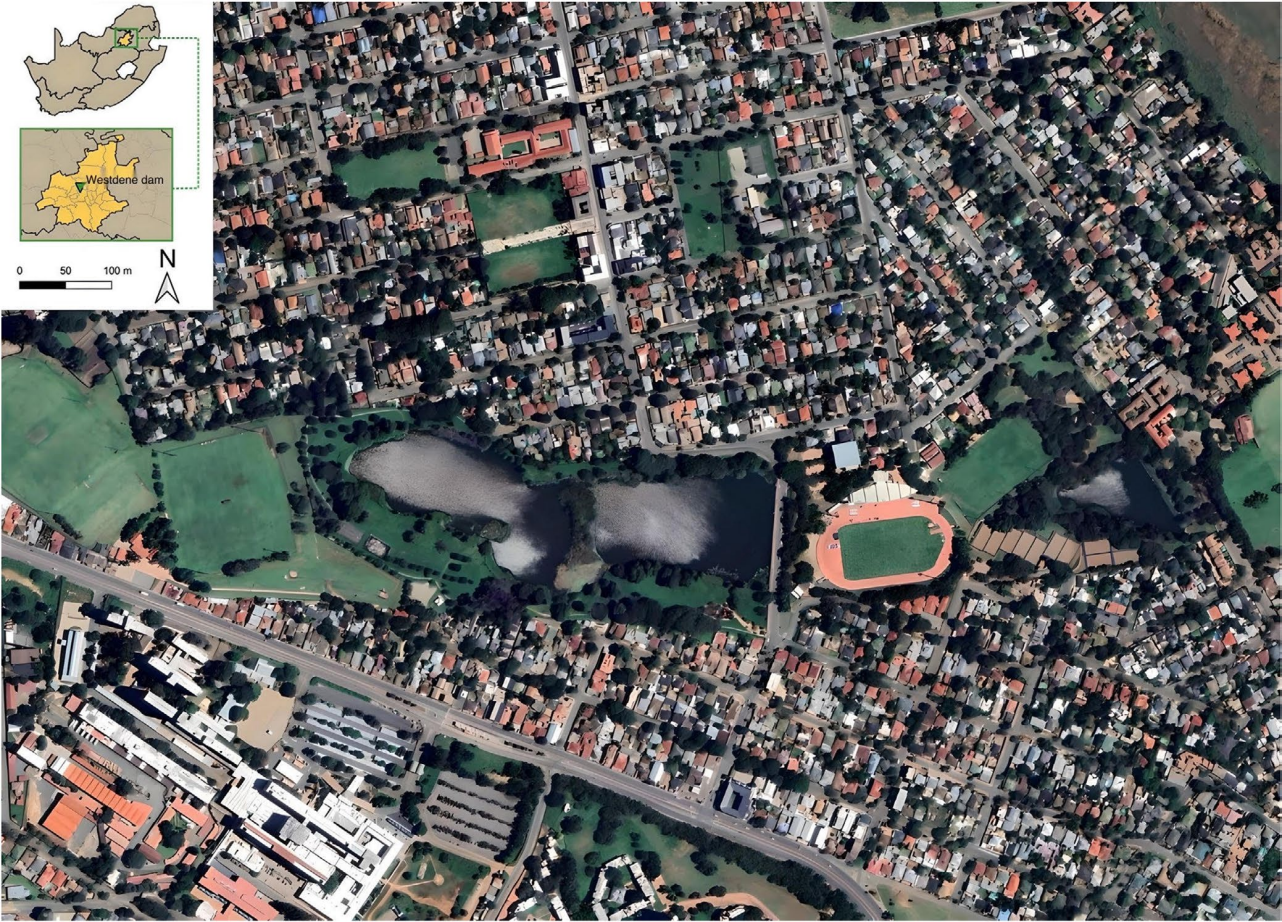
Map of Westdene dam, located within a residential area in Johannesburg (Gauteng, South Africa). The map illustrates the spatial position of the dam in relation to the surrounding urban landscape (Google Earth 2025). Coordinates of sampling sites are listed in S1.

The study area is the Westdene Dam, an artificial waterbody in the west of Johannesburg that is located entirely within a residential area (Figure 1). The dam was constructed in the 1930s by the City of Johannesburg Municipality for recreational purposes such as canoeing and fishing, but it also provides ecological services such as stormwater retention, flood control and filtering of pollutants. The dam covers an area of 0.081 km^2^ and has a volume of 153,900 m^3^. Some of its effluent directly originates from stormwater drains within the residential area (Abiye 2015).

### 2.2 Sampling

The water samples were collected from five sites around Westdene Dam (Figure 1, Figure S1 and Table S1). At each site, to collect a representative eDNA sample and minimise biases in the eDNA recovery rate based on depth (Takahashi et al. 2023), five samples were collected in autoclaved 1 L Schott bottles, four at the surface and one close to the sediment. Samples were immediately stored on ice on‐site, transported to the African Centre for DNA Barcoding at the University of Johannesburg and stored in a freezer at −15°C until being filtered. The collection of water samples was performed with prior authorisation from Johannesburg City Parks and Zoo (https://www.jhbcityparksandzoo.com/) and in accordance with University of Johannesburg regulations.

### 2.3 Filtering of Samples

The water samples were thawed and then filtered using 1.2 μm cellulose nitrate (CN) filters (Sartorius Stedim Biotech, Germany) to capture eDNA contained in the water. This filter size was selected since most eDNA of the plant origins occurs within cells, and the CN filter with a pore size of ~1.2 μm has successfully retrieved eDNA from aquatic habitats in similar studies on plant communities (Hunter et al. 2019; Wilcox et al. 2015, 2013; Bozdogan et al. 2025). Due to the high turbidity of the water samples, two filter papers per 1 L bottle were used for surface water, and four for near‐sediment water. The filter papers were placed into sterile 50 mL centrifuge tubes, which were stored at −20°C until the DNA was extracted within 24 h.

### 2.4 DNA Extraction and Amplification

Environmental DNA was extracted from the filter papers using the CTAB method (Doyle and Doyle 1987). The *rbcLa* region of the chloroplast gene *rbcL* was selected for sequencing due to its extensive reference sequence database, the universal nature of its primers among flowering plants, and the high discriminatory power at the genus level, which makes it particularly suitable for short‐read sequencing (Rattray et al. 2024; Botha et al. 2023; Reddy et al. 2022; Maloukh et al. 2017). This marker was PCR‐amplified at the Canadian Centre for DNA Barcoding (CCDB) using 10.5 μL of a master mix containing platinum *Taq* mix and primers rbcLaF_t1 (TGT AAA ACG ACG GCC AGT ATG TCA CCA CAA ACA GAG ACT AAA GC) and MrbcL‐163R_t1 (CAG GAA ACA GCT ATG ACC GGT CCA YAC AGY BGT CCA KGT ACC), as well as 2 μL of DNA template, with an annealing temperature of 55°C and 60 amplification cycles, following Hausmann et al. (2021). Prior to sequencing, the PCR products were visualised using pre‐cast 2% agarose E‐Gels (ThermoFisher, USA).

Amplicons for each sample were tagged with IonCode universal molecular identifiers (UMIs) (ThermoFisher Scientific, USA) and normalised to 1 ng/μL. Then, BluePippin (Sage Science, USA) and a pre‐cast 2% agarose cassette were used for the size selection step. A purity analysis of the PCR products was performed using a Bioanalyzer (Agilent, USA). Purified PCR products were quantified using the Qubit fluorometer (Invitrogen, USA), with the Qubit HS reagent prepared as per the manufacturer's instructions. The 200 bp Ion Chef setup was used to prepare libraries prior to sequencing. The libraries were then sequenced using the Ion Torrent S5 System sequencing platform (ThermoFisher Scientific, USA) using 600 bp chemistry and a 520 chip at the Canadian Centre for DNA Barcoding.

### 2.5 Bioinformatic Analyses

An initial quality control of the generated raw reads was performed using FastQC (http://www.bioinformatics.bbsrc.ac.uk/projects/fastqc/). Adapter sequences and amplification primers were removed using Cutadapt v.1.18 (Martin 2011). The adapter‐trimmed reads were then processed using the DADA2 v.1.26 R package (Callahan et al. 2016). For this purpose, first, all reads with an expected error rate (Edgar and Flyvbjerg 2015) exceeding the recommended maximum value of 2 were filtered. Then, the error profile of deprecated sequences was predicted using the DADA2 machine‐learning error prediction model. This model predicts the probability of erroneous nucleotide transitions based on the quality scores of sequences and the observed transition rate from each sequencing run. The default parameters for this step remained unchanged from the recommended settings for Ion Torrent platforms available at the developer's website https://benjjneb.github.io/dada2/faq.html. After that, the quality‐filtered sequences were dereplicated into unique sequencing features, and Amplicon Sample Variants (ASVs) were inferred. Following these steps, all potential chimeric ASVs were removed using the chimera detection method implemented in the same package.

For taxonomic rank assignment, the mined local reference sequence database for *rbcLa* was downloaded from the NCBI nucleotide database using Entrez Direct in May 2025 (Kans 2024). The NCBI nucleotide database was selected because it has the largest number of *rbcLa* reference sequences compared to other available databases. This is possibly because the majority of researchers tend to favour lodging their sequence data only with NCBI rather than submitting them to Barcode of Life Data systems (BOLD), due to NCBI's less stringent metadata requirement (Rattray et al. 2024). In addition, sequences submitted to BOLD are automatically fed into NCBI, making NCBI particularly suitable for the selected marker (Rattray et al. 2024).

Each reconstructed ASV was searched against the mined local reference sequence database using blast v.2.15 (Altschul et al. 1990), and the best five matches for each query sequence were reported. Blast parameters were set to their default values except for percentage identity, which was set to a minimum of 98% similarity. A consensus Last Common Ancestor (LCA) taxonomy rank was assigned to each ASV based on the matches using BASTA v1.3.2.3 (Kahlke et al. 2019). Briefly, BASTA utilises the tabular blast output files and assigns the taxonomic rank of each reconstructed ASV to the lowest rank shared among reported matches. When the assignment of a consensus taxonomic rank based on a user‐defined number of best matches is not possible, mainly due to an incomplete reference database, only the best match for each ASV can be reported, as long as the length of the match is longer than 100 bp and the sequence similarity is no less than 98%. In the absence of publicly available records for the focal species or closely related species, the rank assignment in this method is conservative, and higher taxonomic ranks are more likely to be reported in such cases (Kahlke et al. 2019; Webster et al. 2020).

### 2.6 Diversity Estimation and Visualisation

Alpha and beta diversity indices for aquatic and non‐aquatic plants were calculated separately using a combination of Phyloseq v.1.48.0 (McMurdie and Holmes 2013) and vegan v.2.6.6.1 (Oksanen et al. 2020) in R v4.4.0. To minimise bias in the diversity estimates due to unequal sequencing depth, the number of sequences at each site was subsampled to a sequencing depth equal to the minimum sequence depth across all samples.

Three measures of taxonomic richness were calculated for each site: the observed taxonomic diversity, the Shannon index (Shannon and Weaver 1949) and the Simpson index (Simpson 1949). The Kruskal–Wallis rank sum test (Kruskal and Wallis 1952) was used to test for statistically significant differences in selected alpha diversity indices.

For the beta diversity analysis, first, Jaccard's (Jaccard 1908) distance between different sites was calculated in Phyloseq. The implementation of Jaccard's distance in this package can analyse presence–absence data by selecting the binary flag. Then, statistically significant differences in the distance matrices between sites and between collection sites and sample sources (i.e., surface water vs. near‐sediment water) were tested using the adonis2 function in the vegan package with 99,999 permutations.

To investigate the relationship between geographical distance that separates sampling sites and dissimilarity in plant communities, first, a Mantel's Test (Mantel 1967) between Jaccard's distance and geographic distance was performed in vegan. Then, a distance decay analysis was performed using the betapart v.1.6 R package (Baselga and Orme 2012). The statistical significance of correlations between the two matrices was tested using 99,999 permutations.

## 3 Results

The Ion Torrent S5 sequencing run produced a total of 2,298,267 *rbcLa* reads. On average, each sample produced 229,211 reads. Cutadapt identified the presence of an amplification primer in 2,292,109 reads, confirming the successful amplification of the target marker. The remaining reads that were not flanked by an amplification primer were discarded.

**FIGURE 2:**
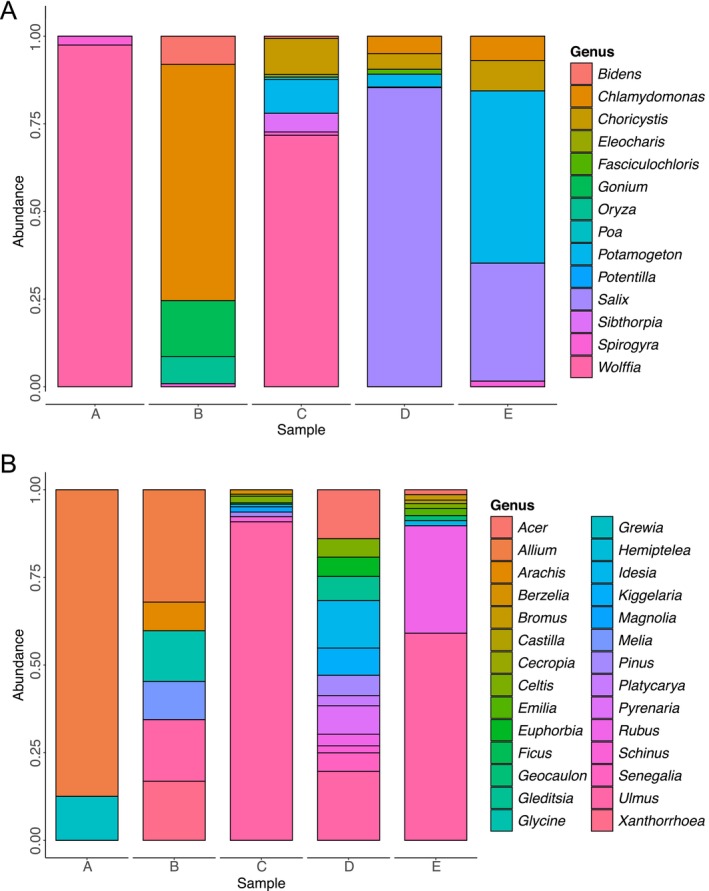
Community composition of aquatic (A) and terrestrial (B) plant genera at Westdene Dam. Sites A, B, C, D & E are sampling sites around the perimeter of the dam. The values on the *Y*‐axis are standardised abundance for each ASV (ASV/∑ASV).

The DADA2 pipeline dereplicated quality‐filtered sequences into 1,133,224 unique sequences. After removing potential chimeric sequences, 1001 ASVs were used for downstream taxonomic rank assignments (Table S2). Among assembled ASVs, 47 unique taxonomic ranks were assigned at the family level, 42 unique ranks at the generic level and only 13 unique ranks at the species level. The remaining ASVs could only be assigned to higher taxonomic ranks, pointing to the incomplete reference sequence database for these plant species (Table S3). In total, putative species belonging to eight aquatic genera (*Chlamydomonas* sp., *Choricystis* sp., *Eleocharis* sp., *Fasciculochloris* sp., *Gonium* sp., *Potamogeton* sp., *Spirogyra* sp., *Wolffia* sp.), three semi‐aquatic (*Bidens* sp., *Oryza* sp., *Salix* sp.) and 31 non‐aquatic genera were identified (Figure 2A,B).

Among ASVs that were assigned a taxonomic rank at the species level, four Alien and Invasive Species (AIS) listed in the South Africa National Environmental Management Biodiversity Act (NEM:BA) list of AIS 2020 were detected. These were *Melia azedarach* L. (NEM: BA category 3), *Tipuana tipu* (Benth.) Kuntze (NEM:BA category 3), *Morus alba* L. (NEM:BA category 3) and *Ricinus communis* L. (NEM:BA category 2). However, since the taxonomic rank assignment of *R. communis* was based on a single direct unpublished record in NCBI, accession number MN099014.1, that was nested within a group of *Salix* species, misidentification during NCBI submission could not be completely ruled out; thus, this species was not further reported.

**FIGURE 3:**
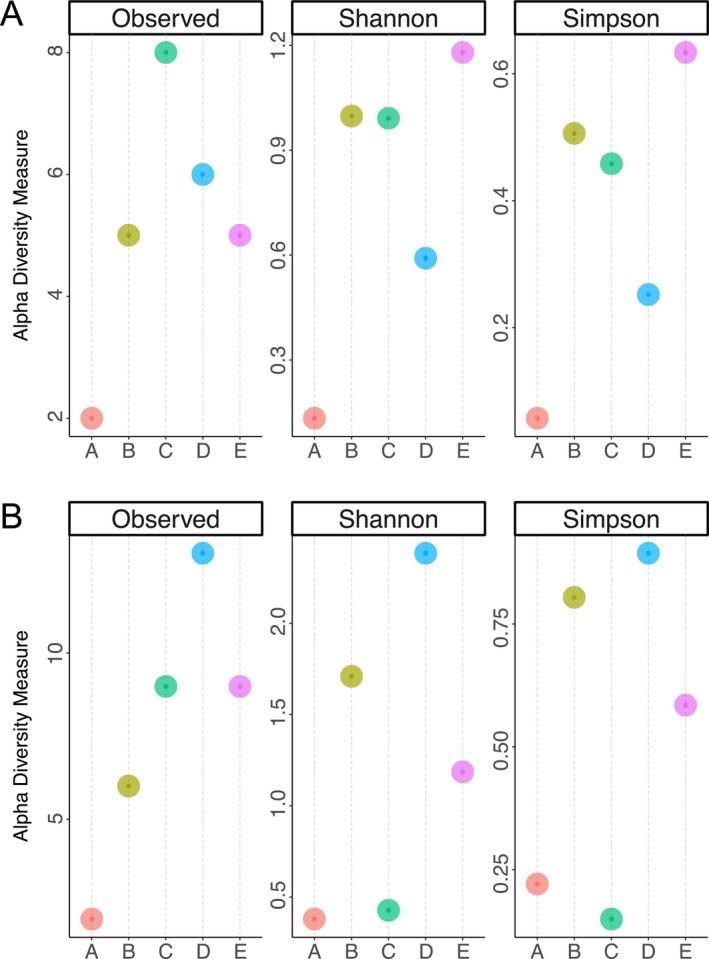
Alpha diversity of aquatic (A) plant and terrestrial (B) community composition using Observed, Shannon and Simpson alpha diversity indices for samples. Labels A, B, C, D and E refer to the sites.

Observed, Shannon and Simpson alpha diversity indices ranged from 1 to 7, 0 to 1.2, and 0 to 0.6 in aquatic species, and 1 to 12, 0 to 2.23 and 0 to 0.87 in non‐aquatic species. In both groups, none of the estimated alpha diversity measures varied significantly between sites, as revealed by the results of the Kruskal–Wallis rank sum test. Similarly, there were no significant differences in indices between surface water and near‐sediment water samples (Table S4, Figure 3A,B).

The *adonis2* test results showed that Jaccard's measure of beta diversity in aquatic plants was just non‐significant (*p =* 0.05) between sites, and it was statistically insignificant (*p =* 0.07) between surface and near‐sediment water samples (Table S5). In contrast, a statistically significant difference (*p =* 0.01) in beta diversity between surface and near‐sediments was observed for non‐aquatic plants, but not between sites (*p =* 0.7) (Figure 3, Table S6).

The results of both Mantel's test and distance decay analysis (Table S7) were statistically insignificant, with a *p*‐value of 0.7 and 0.3 for aquatic plants, and 0.8 and 0.3 for non‐aquatic plants, showing a lack of correlation between geographical distance and plant community dissimilarity in the studied location.

## 4 Discussion

The assessment of biodiversity through environmental DNA (eDNA) metabarcoding is a powerful tool for the establishment, implementation and monitoring of ecosystem management strategies in urban areas. In this study, we used eDNA metabarcoding to survey community‐level plant biodiversity in an urban dam in Johannesburg, South Africa's largest city.

The metabarcoding of the *rbcLa* marker identified three invasive species in the surveyed habitat, which are *Melia azedarach* , *Tipuana tipu* and *Morus alba* . However, on the day of sample collection, none of these species were visually identifiable. A search of the GBIF (GBIF.org) plant occurrence database conducted in May 2025 confirmed that *M. azedarach* (Joly et al. 2014, 2016; Goeau et al. 2017; Affouard et al. 2017; Richardson and Potgieter 2024; GBIF.org 2025a; GBIF.org 2025b; GBIF. org 2025c; GBIF.org 2025d; GBIF.org 2025e; GBIF.org 2025f; GBIF.org 2025g; GBIF.org 2025h; GBIF.org 2025i; GBIF.org 2025j; GBIF.org 2025k; GBIF.org 2025l; GBIF.org 2025m; GBIF.org 2025n; GBIF.org 2025o; GBIF.org 2025p; GBIF.org 2025q; Ranwashe 2025; European Bioinformatics Institute 2025), *T. tipu* (Richardson and Potgieter 2024; GBIF.org 2025a; GBIF.org 2025b; GBIF.org 2025c; GBIF.org 2025d; GBIF.org 2025e; GBIF.org 2025f; GBIF.org 2025g; GBIF.org 2025h; GBIF.org 2025i; GBIF.org 2025j; GBIF.org 2025k; GBIF.org 2025l; GBIF.org 2025m; GBIF.org 2025n; GBIF.org 2025o; GBIF.org 2025p; GBIF.org 2025q; Bijmoer et al. 2024; Ranwashe 2025) and *M. alba* (Richardson and Potgieter 2024; GBIF.org 2025a; GBIF.org 2025b; GBIF.org 2025c; GBIF.org 2025d; GBIF.org 2025e; GBIF.org 2025f; GBIF.org 2025g; GBIF.org 2025h; GBIF.org 2025i; GBIF.org 2025j; GBIF.org 2025k; GBIF.org 2025l; GBIF.org 2025m; GBIF.org 2025n; GBIF.org 2025o; GBIF.org 2025p; GBIF.org 2025q) have already been reported from Johannesburg. This demonstrates the effectiveness of eDNA in the detection of invasive species, whose presence might otherwise go unnoticed during visual surveys.

The early detection of potentially invasive species in non‐native habitats enables ecosystem managers to implement prompt control and management measures to eradicate or contain their spread and minimise negative impacts on native species before they can fully establish themselves. Populations of introduced species typically remain at low densities for years prior to becoming invasive (Kelly et al. 2021), and molecular monitoring of ecosystems is thus a valuable means of documenting their presence during the early stage of the invasion process.

Among the identified invasive species in this study, *Melia azedarach* , commonly known as syringa, is an alien invasive tree of conservation concern in native South African ecosystems. It is listed as a category 1b species (i.e., it needs to be controlled) in the South Africa National Environmental Management: Biodiversity Act or, if found in urban areas, a category 3 species that cannot be propagated, traded, or planted further. This species is highly invasive in the warm eastern and northern regions of the country, and it is considered one of the top 10 most invasive plant species in terms of the areas it covers (Van Wilgen and Wilson 2018). When this species invades an urban area, it causes alterations in vegetation structure and plant composition, facilitating the spread of the obligate hemiparasitic mistletoe, *Viscum album* (Bhatt et al. 2021). Mistletoe has a long‐lasting impact on the natural habitats in which it establishes itself. It has been associated with lowering taxonomic richness, reducing the tree diameter and altering the seedling and adult tree communities (Bhatt et al. 2021; Silva et al. 2024).

*Tipuana tipu* (tipa) is a leguminous tree from South America that has become invasive in regions of Australia and South Africa characterised by high concentrations of phosphorus in the soil (Trudgen et al. 2023). Its ornamental value, its usage as a source for honey production and its economic significance in the timber and fodder industries have contributed to its human‐mediated spread across the globe (dos Santos Pereira and de Aquino Neto 2003).

The remaining species, *M. alba* , is a widespread invader known to alter local ecosystems. In North America, *M. alba* crosses with the indigenous species, such as *Morus rubra* , and gradually replaces this native species across its distribution area (Hassan et al. 2018). It is currently unknown whether it could have a similar impact on local species in South Africa.

In the Westdene Dam habitat, several taxa of aquatic plants have been identified. For instance, species belonging to the genus *Potamogeton* are widely used to assess the integrity of aquatic ecosystems in response to multi‐source pollution (Harguinteguy et al. 2016). Bertrand et al. 2019, conducted a review of the efficiency of *Potamogeton pusillus* for environmental risk assessment in aquatic systems and found high sensitivity of this species to metal pollutants such as lead, aluminium, arsenic, boron and mercury. The *Wolffia* sp., another aquatic species identified from the area, is recognised as a bioindicator of cadmium contamination (Parmar et al. 2016). Pereira et al. (2019), reported the sensitivity of *Wolffia brasiliensis* to various herbicides, suggesting the use of this aquatic plant as a bioindicator for the risk assessment of herbicides in aquatic ecosystems.

A number of algae were also detected. Among these, *Chlamydomonas* sp., *Choricystis* sp. and *Eleocharis* sp. are indicators of aquatic pollution (Zaghloul et al. 2020; O'Neill and Rowan 2022), and their diversity can reflect changes in environmental conditions due to anthropogenic activities, including changes in land use. Their presence in aquatic ecosystems could suggest an increase in the concentration of nutrients such as NO_3_^−^, NH_4_^+^, PO_4_^3−^, Cl^−^ and SO_4_^−^ (Roy 2022; Dubey et al. 2022). The presence of *P. pusillus* , *Eleocharis* sp. and *Wolffia* sp. in and around the City of Johannesburg was further substantiated by GBIF observational data. While earlier studies of the water quality at the Westdene Dam indicated high concentrations of ammonia, phosphate and metals such as nickel, cadmium, cobalt and lead (Masetle 2013), establishing a direct link between the taxonomic diversity of aquatic plants and pollution levels requires repeating molecular surveys similar to the one conducted here across multiple urban aquatic habitats with contrasting levels of water pollution and plant biodiversity, and this remains an interesting subject for future research (Delmail 2014; Stefanidis et al. 2021; Hou et al. 2024).

**FIGURE 4:**
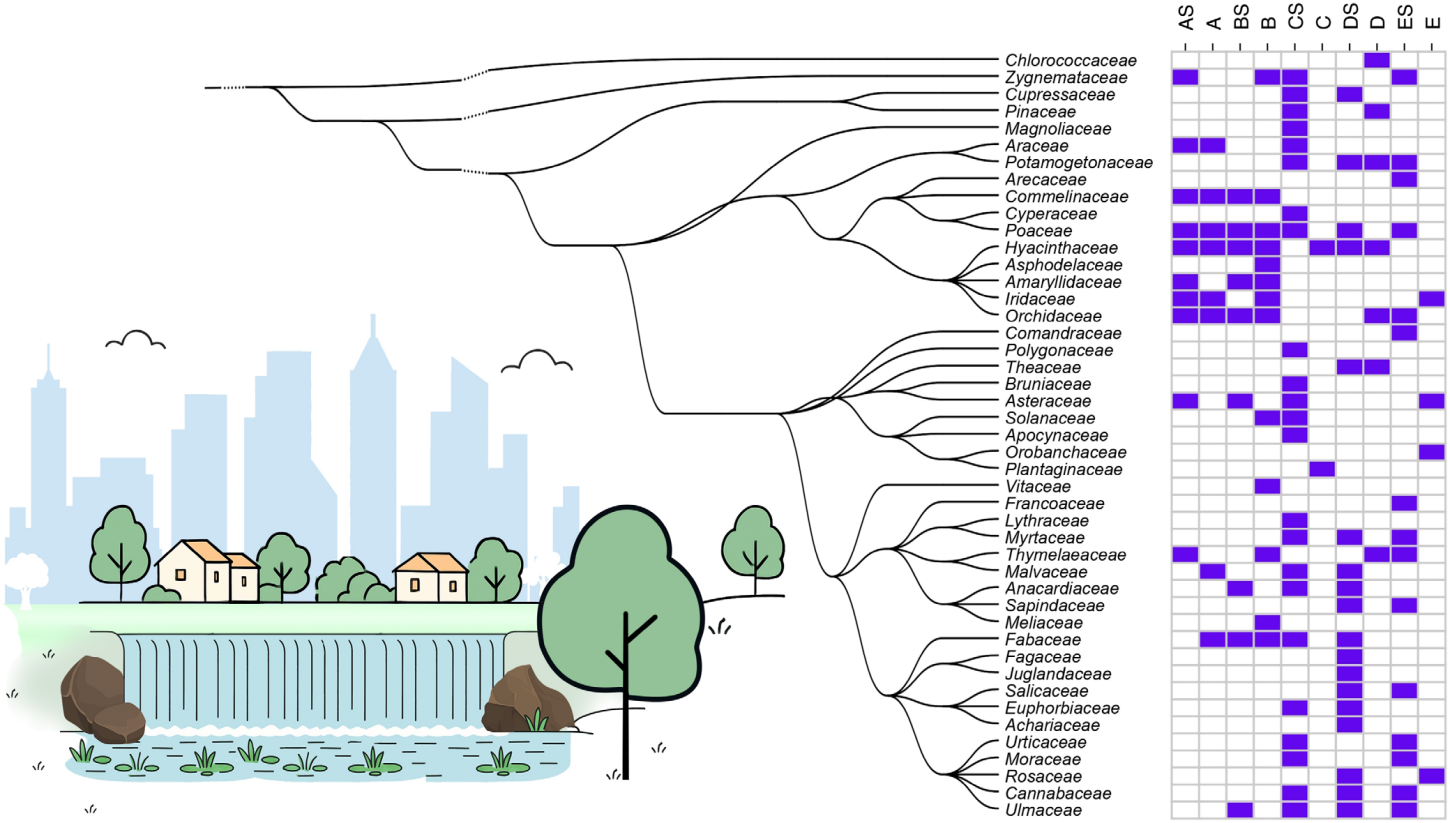
Presence and absence clustering phylogenetic tree of the plant families in the ten samples collected from Westdene Dam. Blue indicates the presence of families at a particular site, while white represents their absence. Labels A, AS, B, BS, C, CS, D, DS, E & ES refer to the samples. The letter ‘S' in sample labels refers to near‐sediment samples; The topology is based on the consensus NCBI taxonomy placement of the reported families reconstructed using the phyloT web server (https://phylot.biobyte.de) (Letunic and Bork 2021).

A statistically borderline insignificant yet noticeable variation in plant biodiversity was observed between sites and between taxonomic diversity reconstructed from surface and near‐sediment waters. While some species, such as members of Hyacinthaceae, were present at the majority of sites (found in: A, AS, B, BS, C and E), others, such as members of Polygonaceae, Chlorococcaceae and Vitaceae, were only observed at one site (Figure 4).

To fully represent ecological biodiversity, a molecular survey must take into account compositional variation within a given site. When the distribution of biodiversity across a landscape is incorrectly assumed to be homogeneous, surveys based on unrepresentative samples typically collected from a few locations cannot reflect the true level of diversity in that area (Drummond et al. 2021). Our results also emphasise the importance of obtaining samples from various sources (including different water depths) to better describe local biodiversity.

Sample DS was dominated by Salicaceae (the willow family), which is confirmed by a high abundance of *Salix babylonica* L. Although this species was visually identified on the day of sample collection, we were unable to identify it to the species level using the molecular survey technique. This highlights one of the important limitations of using eDNA, which is achieving species‐level identifications for challenging plant groups in geographical regions whose species are underrepresented in DNA reference collections, such as South Africa. In these ecosystems, this method primarily allows for the reconstruction of plant community composition at higher taxonomic rankings, that is family and genus taxonomic levels, due to incomplete reference databases.

This conclusion is consistent with that of a recent study conducted by Rattray et al. (2024), which showed that almost all plant families in South Africa have been underbarcoded, with the Zamiaceae being the only exception, emphasising the lack of coverage of South African flora in the reference databases. This underrepresentation of native South African species remains a significant constraint to eDNA‐based studies, echoing broader challenges in biodiversity research on the African continent (Serite et al. 2023; Vasar et al. 2023; von der Heyden 2023). It underscores the need for collaborative efforts, standardised procedures and the continuous expansion of reference databases to unlock the full potential of eDNA metabarcoding in improving biodiversity conservation and management efforts in South Africa and beyond.

Traditional surveying techniques for plant biodiversity assessments are typically time‐consuming, labour‐intensive and limited in spatial extent (Lønborg et al. 2021). Field sampling factors such as inaccessibility due to dense vegetation, the rapid growth and unnoticed movement of invasive species and seasonal changes make it difficult to ensure comprehensive and consistent sampling across large geographic areas (Jakubauskas et al. 2002). Furthermore, traditional surveying methods rely heavily on taxonomic expertise—a skill that is becoming increasingly scarce, as fewer individuals are trained in classical plant taxonomy (Engel et al. 2021).

Molecular techniques, on the other hand, enable non‐specialists to collect meaningful ecological data (such as community composition) more efficiently and without requiring specialised taxonomic expertise. However, this method is currently constrained by incomplete reference libraries and the comparatively high costs of eDNA sequencing. Including additional gene regions or complete chloroplasts that may be required for greater identification power may not be financially feasible, mainly for small and medium‐sized laboratories in developing countries. Therefore, there is a trade‐off between these two approaches, and the specific research question and available resources should guide the choice of technique.

While a wide range of taxonomic groups, including some molluscs, chordates and various zooplanktons and phytoplanktons (Santos and Ferreira 2020), have traditionally been used to monitor the ecological state of ecosystems, the compositional diversity of macrophyte communities has only received the same level of attention comparatively recently. Coupled with the rapid application and increasing affordability of molecular methods such as eDNA metabarcoding in ecological studies, it provides the scientific community with a new opportunity to effectively monitor the ecological state and the health of urban ecosystems.

## Author Contributions

**N. Nhlengethwa:** conceptualization (equal), data curation (equal), formal analysis (equal), investigation (equal), methodology (equal), project administration (equal), visualization (equal), writing – original draft (equal), writing – review and editing (equal). **R. D. Stewart:** conceptualization (equal), data curation (equal), formal analysis (equal), funding acquisition (equal), investigation (equal), methodology (equal), writing – original draft (equal), writing – review and editing (equal). **A. Emami‐Khoyi:** conceptualization (equal), data curation (equal), formal analysis (equal), investigation (equal), methodology (equal), resources (equal), software (equal), supervision (equal), validation (equal), visualization (equal), writing – original draft (equal), writing – review and editing (equal). **P. R. Teske:** resources (equal), software (equal), writing – original draft (equal), writing – review and editing (equal). **S. Csányi:** writing – review and editing (equal). **M. Heltai:** writing – review and editing (equal). **M. van der Bank:** conceptualization (equal), data curation (equal), formal analysis (equal), funding acquisition (equal), investigation (equal), methodology (equal), project administration (equal), resources (equal), software (equal), supervision (equal), validation (equal), visualization (equal), writing – original draft (equal), writing – review and editing (equal).

## Conflicts of Interest

The authors declare no conflicts of interest.

## Supporting information


**Appendix S1:** ece372196‐sup‐0001‐AppendixS1.pdf.

**Table S2:** ece372196‐sup‐0002‐TableS2.pdf.

**Table S3:** ece372196‐sup‐0004‐TableS3.xlsx.

**Table S4:** ece372196‐sup‐0005‐TableS4.pdf.

**Table S5:** ece372196‐sup‐0006‐TableS5.pdf.

**Table S6:** ece372196‐sup‐0007‐TableS6.pdf.

**Table S7:** ece372196‐sup‐0008‐TableS7.pdf.

## Data Availability

The raw sequences for this project have been submitted to NCBI Sequence Read Archive (SRA) under Bioproject accession number PRJNA1163254 https://www.ncbi.nlm.nih.gov/sra/PRJNA1163254.
