# Supplementary material for: An eDNA Survey of Plant Biodiversity in a Local Dam Within South Africa's Largest City
Source: Ecol Evol. 2025 Sep 28;15(10):e72196. doi: 10.1002/ece3.72196 (PMC12476927; doi:10.1002/ece3.72196)
Supplement: Supplementary file 1 — Appendix S1: ece372196‐sup‐0001‐AppendixS1.pdf. [file ECE3-15-e72196-s007.pdf]

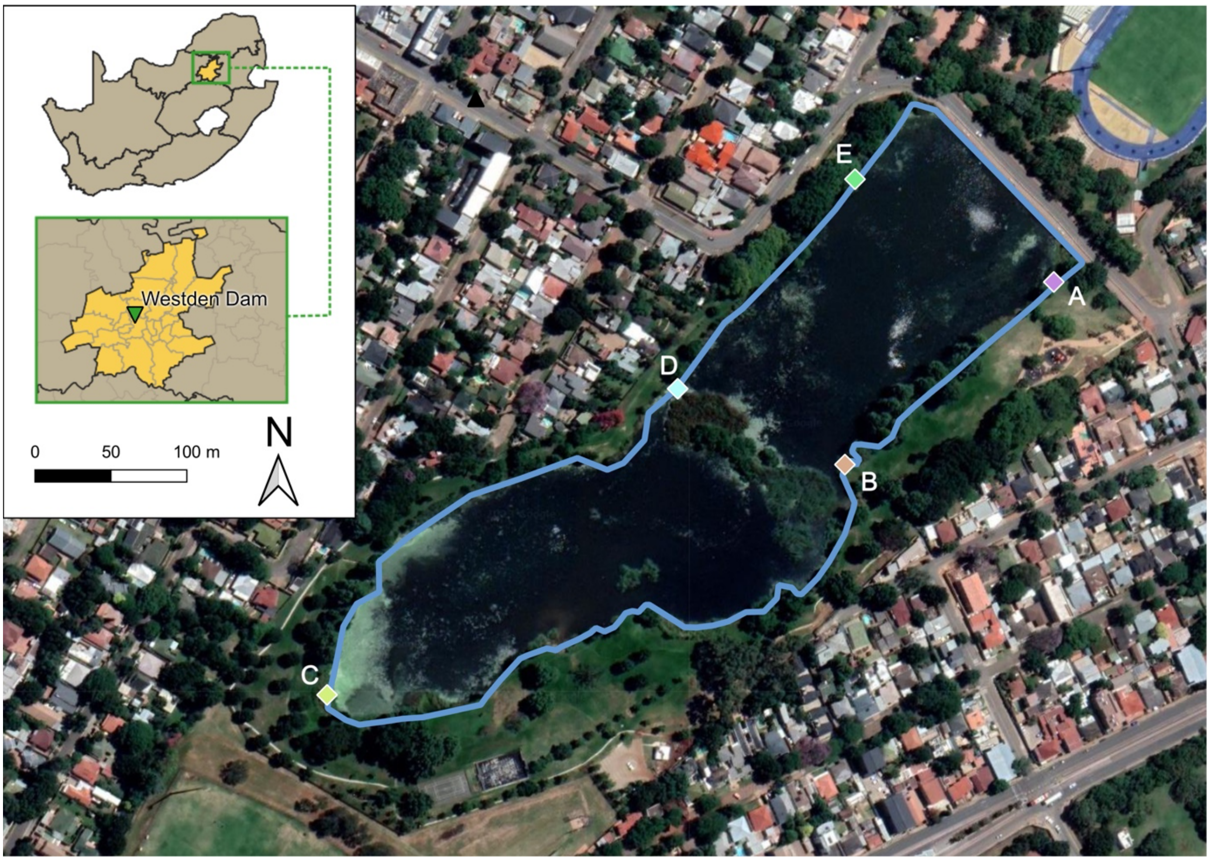

Figure S1: Map of Westdene dam showing exact locations of sampling sites.

Table S1: Sample site coordinates and sampling dates

Site	Coordinates	Date
A	26 10 47 S 27 59 29 E	09/2022
C	26 10 53 S 27 59 22 E	09/2022
D	26 10 56 S 27 59 12 E	09/2022
E	26 10 49 S 27 59 19 E	09/2022
F	26 10 45 S 27 59 24 E	09/2022
