# Supplementary material for: An eDNA Survey of Plant Biodiversity in a Local Dam Within South Africa's Largest City
Source: Ecol Evol. 2025 Sep 28;15(10):e72196. doi: 10.1002/ece3.72196 (PMC12476927; doi:10.1002/ece3.72196)
Supplement: Supplementary file 2 — Table S2: ece372196‐sup‐0002‐TableS2.pdf. [file ECE3-15-e72196-s004.pdf]

Table S2: Summary of sequencing depth

	input	filtered	denoised	nonchim
A	247516	243082	240910	133763
AS	211358	208312	206037	111814
B	279148	273286	270466	135751
BS	285036	280896	278639	158189
C	123635	121079	121002	120225
CS	193798	190308	187969	115836
D	172656	170183	168163	72148
DS	226391	222522	221057	131932
E	303610	301874	299488	117101
ES	248961	243912	241605	108482
Ave	229210.9	225545.4	223533.6	120524.1
