# Supplementary material for: An eDNA Survey of Plant Biodiversity in a Local Dam Within South Africa's Largest City
Source: Ecol Evol. 2025 Sep 28;15(10):e72196. doi: 10.1002/ece3.72196 (PMC12476927; doi:10.1002/ece3.72196)
Supplement: Supplementary file 4 — Table S4: ece372196‐sup‐0005‐TableS4.pdf. [file ECE3-15-e72196-s001.pdf]

Table S4-a: Alpha diversity measures for aquatic plant community.Values rounded to three digits.

Sample	Observed	Shannon	Simpson
AS	2	0.124	0.053
A	1	0.000	0.000
CS	2	0.492	0.313
C	4	0.996	0.569
DS	7	0.803	0.384
D	1	0.000	0.000
ES	4	0.189	0.075
E	4	1.165	0.646
FS	5	1.203	0.642

Table S4-b: Alpha diversity measures for aquatic plant community.Values rounded to three digits.

Samples	Observed	Shannon	Simpson
AS	1	0.000	0.000
CS	3	1.061	0.641
C	3	1.04	0.626
DS	8	0.356	0.126
ES	12	2.239	0.873
E	1	0.000	0.000
FS	7	0.535	0.208
F	2	0.236	0.119
