# Supplementary material for: An eDNA Survey of Plant Biodiversity in a Local Dam Within South Africa's Largest City
Source: Ecol Evol. 2025 Sep 28;15(10):e72196. doi: 10.1002/ece3.72196 (PMC12476927; doi:10.1002/ece3.72196)
Supplement: Supplementary file 5 — Table S5: ece372196‐sup‐0006‐TableS5.pdf. [file ECE3-15-e72196-s003.pdf]

Table S5-a: Adonis2 PERMANOVA results using Jaccard Distance by site for terrestrial plant community

```
adonis2(formula = jac_dist ~ sample_data(ps.rarefied)$site)
         Df SumOfSqs      R2      F Pr(>F)
Model     4   1.6951 0.45875 0.8476      1
Residual  4   2.0000 0.54125
Total     8   3.6951 1.00000
Permutation test for adonis under reduced model
Permutation: free

Number of permutations: 99999
```

Table S5-b: Adonis2 PERMANOVA results using Jaccard Distance by source (surface water vs near-sediment samples) for terrestrial plant community

```
adonis2(formula = jac_dist ~ sample_data(ps.rarefied)$source)
         Df SumOfSqs      R2      F Pr(>F)
Model     1   0.6532 0.17677 1.5031  0.028 *
Residual  7   3.0419 0.82323
Total     8   3.6951 1.00000
---
Signif. codes:  0 ‘***’ 0.001 ‘**’ 0.01 ‘*’ 0.05 ‘.’ 0.1 ‘ ’ 1
Permutation test for adonis under reduced model
Permutation: free
Number of permutations: 99999
```
