# Supplementary material for: An eDNA Survey of Plant Biodiversity in a Local Dam Within South Africa's Largest City
Source: Ecol Evol. 2025 Sep 28;15(10):e72196. doi: 10.1002/ece3.72196 (PMC12476927; doi:10.1002/ece3.72196)
Supplement: Supplementary file 6 — Table S6: ece372196‐sup‐0007‐TableS6.pdf. [file ECE3-15-e72196-s005.pdf]

Table S6-a: Adonis2 PERMANOVA results using Jaccard Distance by site for aquatic plant community

adonis2(formula = jac_dist ~ sample_data(ps.rarefied)$site)

	Df	SumOfSqs	R2	F	Pr(>F)	
Model	4	1.9687	0.62779	1.6866	0.05	*
Residual	4	1.1672	0.37221			
Total	8	3.1359	1.00000			

Signif. codes: 0 ‘***’ 0.001 ‘**’ 0.01 ‘*’ 0.05 ‘.’ 0.1 ‘ ’ 1

Permutation test for adonis under reduced model

Permutation: free

Number of permutations: 99999

Table S6-b: Adonis2 PERMANOVA results using Jaccard Distance by source (surface water vs near-sediment samples) for aquatic plant community

adonis2(formula = jac_dist ~ sample_data(ps.rarefied)$source)

	Df	SumOfSqs	R2	F	Pr(>F)
Model	1	0.3094	0.09867	0.7663	0.674
Residual	7	2.8265	0.90133		
Total	8	3.1359	1.00000		

Number of permutations: 99999
