# Supplementary material for: An eDNA Survey of Plant Biodiversity in a Local Dam Within South Africa's Largest City
Source: Ecol Evol. 2025 Sep 28;15(10):e72196. doi: 10.1002/ece3.72196 (PMC12476927; doi:10.1002/ece3.72196)
Supplement: Supplementary file 7 — Table S7: ece372196‐sup‐0008‐TableS7.pdf. [file ECE3-15-e72196-s002.pdf]

Table S7-a : Summary of distance decay analysis between beta diversity distance and geographic distance for aquatic plant community

```
$model
Nonlinear regression model
  model: ydis ~ a * exp(-b * x)
   data: parent.frame()
       a        b
   0.0378 -284.9431
 residual sum-of-squares: 1.133

Number of iterations to convergence: 31
Achieved convergence tolerance: 1.49e-08

$model.type
[1] "exponential"

$y.type
[1] "dissimilarities"

$first.parameter
       a
0.9621967

$second.parameter
       b
-284.9431
```

$aic

[1] -16.35993

$pseudo.r.squared

[1] 0.006225729

$p.value

[1] 0.6276363

attr(,"class")

[1] "decay"

Table S7-b: Summary of distance decay analysis between betadiversity distance and geographic distance for terrestrial plant community

```
$model
Nonlinear regression model
  model: ydis ~ a * exp(-b * x)
   data: parent.frame()
        a          b
   0.02007 -309.56895
 residual sum-of-squares: 0.4979

Number of iterations to convergence: 12
Achieved convergence tolerance: 1.49e-08
```

```
$model.type
[1] "exponential"

$y.type
[1] "dissimilarities"

$first.parameter
        a
0.9799341

$second.parameter
       b
-309.569

$aic
[1] -45.94633

$pseudo.r.squared
[1] 0.03761227

$p.value
[1] 0.3199232

attr(,"class")
[1] "decay"
```
